# Development of an efficient *Tef-1α* RNA hairpin structure to efficient management of *Lasiodiplodia theobromae* and *Neofusicoccum parvum*

**DOI:** 10.1038/s41598-021-88422-1

**Published:** 2021-05-05

**Authors:** Omid Nili, Abdolbaset Azizi, Jafar Abdollahzadeh

**Affiliations:** Department of Plant Protection, University of Kurdistan, 66177-15175 Sanandaj, Iran

**Keywords:** Plant biotechnology, Plant breeding, Plant hormones, Plant stress responses, Agricultural genetics, Molecular engineering in plants

## Abstract

*Lasiodiplodia theobromae* and *Neofusicoccum parvum *are serious worldwide-distributed plant pathogenic fungi with a wide host range in tropical and temperate climates. They cause fruit rot, canker, and dieback of twigs in various woody plants. Protection of pruning wounds using fungicides is the prevalent strategy for the management of the diseases caused by these fungi. Chemical control of plant diseases is not environmentally safe and the residues of fungicides are a threat to nature. Furthermore, genetic resources of resistance to plant diseases in woody plants are limited. The aim of this study was to investigate the efficiency of RNA silencing using an efficient hairpin structure based on *Tef-1α* gene for the management of *L. theobromae* and *N. parvum*. Hairpin structure of *Tef-1α* was cloned in *pFGC*5941 binary vector and the recombinant construct was named *pFGC-TEF-d*. Transient expression of *pFGC-TEF-d* using *Agrobacterium* LBA4404 in grapevine (Bidaneh Sefid cv.) and strawberry cultivars (Camarosa and Ventana) led to a reduction in disease progress of *L. theobromae*. The disease reduction in grapevine was estimated by 55% and in strawberries cultivars Camarosa and Ventana by 58% and 93%, respectively. Further analysis of transient expression of *pFGC-TEF-d* in strawberry (Camarosa) shown disease reduction using *Neofusicoccum parvum*. Here we introduce RNAi silencing using *pFGC-TEF-d* construct as an efficient strategy to the management of *L. theobromae* and *N. parvum* for the first time.

## Introduction

Members of Botryosphaeriaceae are important fungi found as endophyte, saprophyte and parasite often on woody plants. These fungi are associated with stem canker, fruit rot, dieback, and blight of blossom and leaves^1^.

*L. theobromae* and *N. parvum* are worldwide distributed botryosphaeriaceous species that cause important diseases in tropical and temperate regions especially on plants subjected to stress^2^. These species with a wide host range are mostly associated with woody plants including economically important fruit trees such as grapevine^3,4^. Over the last decades in the light of global climate change, more stress on plants and changes in pathogen behavior, host–pathogen interactions, and microbial communities, Botryosphaeriaceae members have become more prevalent and received much more attention^5,6^. Moreover, *L. theobromae*, as a well-known Botryosphaeriaceae member, has been reported as a human opportunistic pathogen causing inflammation and lesion on skin^7^, ocular keratitis, and endophthalmitis^8,9^ and onycho-mycosis^10^. Several strategies used to manage these pathogens have mostly focused on the protection of the pruning wound using chemicals and biocontrol agents, avoid wounding of plants, and minimize exposure to stress. Fungicides application and pruning of infected branches are the main methods to the reduction of disease rate and severity^11^. Despite the capability of fungicides in controlling plant pathogens, they can be active a few days after applications^12,56^ and research findings indicated that fungicides have limited effect to protect pruning wounds from *Botryosphaeriaceae members*^13^. Biocontrol is an environmentally safe strategy in integrated pest management of plant diseases^12,14^, especially using some bacteria as biocontrol agents^14^. Although some effective bacteria such as actinobacteria have been reported as biocontrol agents of *L. theobromae* ^14^ and *N. parvum*, the efficacy of biological control agents depends on the climates^16^. Moreover, host trees are basically much more susceptible to Botryosphaeriaceae members under stress condition^57^, which leads to the partial efficiency of biocontrol agents. Thus, to achieve efficient management of these fungi considering novel and alternative methods is an inevitable necessity. RNA silencing as a conserved RNA-mediated gene silencing mechanism is a new and safe strategy that recently has been widely investigated in plant disease management^18^. This is a phenomenon that reduces or stops the expression of a specific gene^19^. In eukaryotic species, gene silencing has an important role in the regulation of gene expression, DNA methylation, and cell immunity against viruses and transposons^20^. However, RNA interference (RNAi) shows the ability for effective control of pests and diseases^21^. In RNA interference (RNAi) technology, the construct contains a complementary strand of mRNA hybridizes with the target sequence which leads to the formation of a double strand RNA (dsRNA) structure. Afterward, the dsRNA or hairpin RNA (hpRNA) is cleaved by Dicer or Dicer-like enzyme to generate 21- to 25-nt siRNAs, which are guiding RNA-induced silencing complexes (RISC)^22^. However, the translation machinery system cannot translate dsRNA and interfere with the translation of complementary mRNA^23^. Transgenic resistant plants developed based on this phenomenon have to produce siRNA using hairpin gene construct containing partially inverted repeat of the gene^24^. Previous studies showed that the inserted hairpin structure confers suitable resistance to homologous sequences (more than 90% homology)^24,25^. De novo siRNA can spread systematically throughout the plant and interferes with plant pathogen target sequence^26^. Application of RNAi for inhibition of plant pathogenic fungi becomes an interest for researchers, especially for filamentous fungi^27,28^. The aim of this research was to design an effective gene construct for the effective and environmentally safe management of *L. theobromae* and *N. parvum* using RNA silencing.

## Results

### Insilico analysis

Blast search of *Tef-1α* in NCBI showed more than 96% similarity between *L. theobromae* isolates. Because of the potentially silencing unwanted genes in host plants expressing hairpin structure and small RNAs, and also potentially off-targeting the human’s genes as an important consumer by passing through the digestive system to the hemolymph and circulatory system, off-target investigation using RNAi scan revealed *L. theobromae Tef-1α* gene (MG192354.1) has no fragment contain necessary homology with human, strawberry and grapevine genome requires for siRNA attack. No hit recognized in humans and grapevine for 10 efficient siRNAs predicted from *pFGC-TEF-d* construct (see Supplementary Table S2 online). Sequence similarity of *Tef-1α* of *L. theobromae* and *Trichoderma atroviride* (MT793743.1) was investigated, and it showed low sequence similarity (55%), which is not enough for induction of silencing against *T. atroviride* as a biocontrol agent.

RNA structure software showed a high degree of predicted secondary structure in mRNA of *Tef-1α* (Supplementary Fig S1 online). However, miRNAFold predicted two microRNAs and shows more possible roles for *Tef-1α*, (Supplementary Fig S2 online). Alignment analysis of *L. theobromae Tef-1α* [MG192354] showed low sequence similarity (72%) with *N. parvum Tef-1α* [JQ772082] (Supplementary Fig S4 online), but in some regions, the similarity was enough to predict a hit for siRNA or microRNA produced by *L. theobromae Tef-1α*-dimer. Red boxes show predicted hit regions (Supplementary Fig S4 online).

### Gene construct

**Figure 1:**
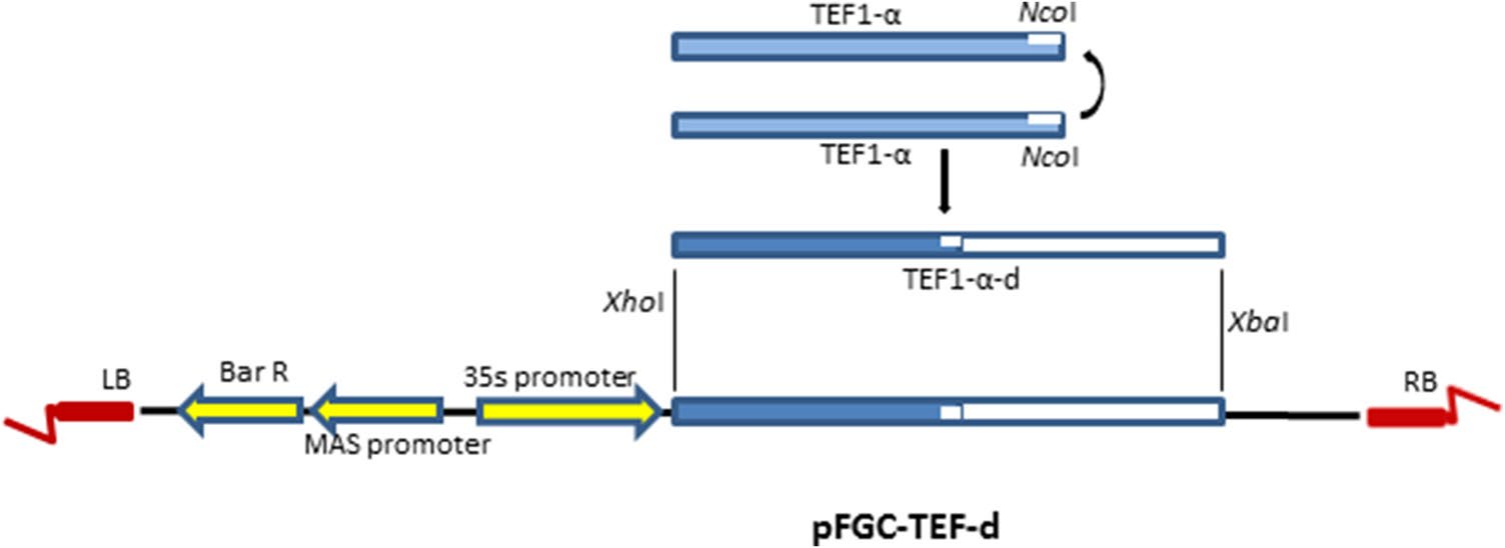
Schematic figure of cloning steps of *Tef-1α* into a binary vector.

PCR amplification of *Tef-1α* gene using specific primers (La.TEF1-α and La.TEF1-α-R) resulted a 316 bp PCR product (Supplementary Fig S3a online). The PCR product was digested using *Nco*I restriction enzyme and purified. The digested product was self-ligated to produce *Tef-1α* dimer (*Tef-1α-d*). After ligation, 600 bp *Tef-1α* dimer was amplified using TEF1-α-F primer (Supplementary Fig S3b online). The amplified dimer was cloned into pTG19-T and the recombinant plasmid (pTG19-T-d) was digested using *Xba*I and *Xho*I enzymes. Moreover, the digested *Tef-1α-d* band was extracted and cloned into *pFGC*5941 binary vector. The ≈600 bp amplified band using clone PCR (Supplementary Fig S3c online) and restriction digestion of recombinant binary vector using *BamH*I enzyme (Supplementary Fig S3d online) confirmed the recombination of *pFGC*5941 and the recombinant plasmid was named *pFGC-TEF-d*. Moreover, the recombinant plasmid was sequenced by Bio Magic Gene Company (Hong Kong, China). Schematic representation of different stages of *pFGC-TEF-d* construction shows in Fig. 1.

### Bioassay and evaluation of gene construct efficiency

Bioassay evaluation on strawberry leaves showed that *L. theobromae* (isolate IRAN 1499C) reacts differentially in two tested cultivars so that three and eight days post-inoculation (dpi) necrosis observed in Camarosa and Ventana cultivars, respectively. Necrosis was developed on grapevine and strawberry leaves five dpi with *N. parvum*.

To evaluate the efficacy of *pFGC-TEF-d* construct on inhibition of *L. theobromae* and *N. parvum*, Agrobacterium cell suspension containing *pFGC-TEF-d* was vacuum infiltrated into leaves and after three days, inoculation with fungal cultures was performed.

**Figure 2:**
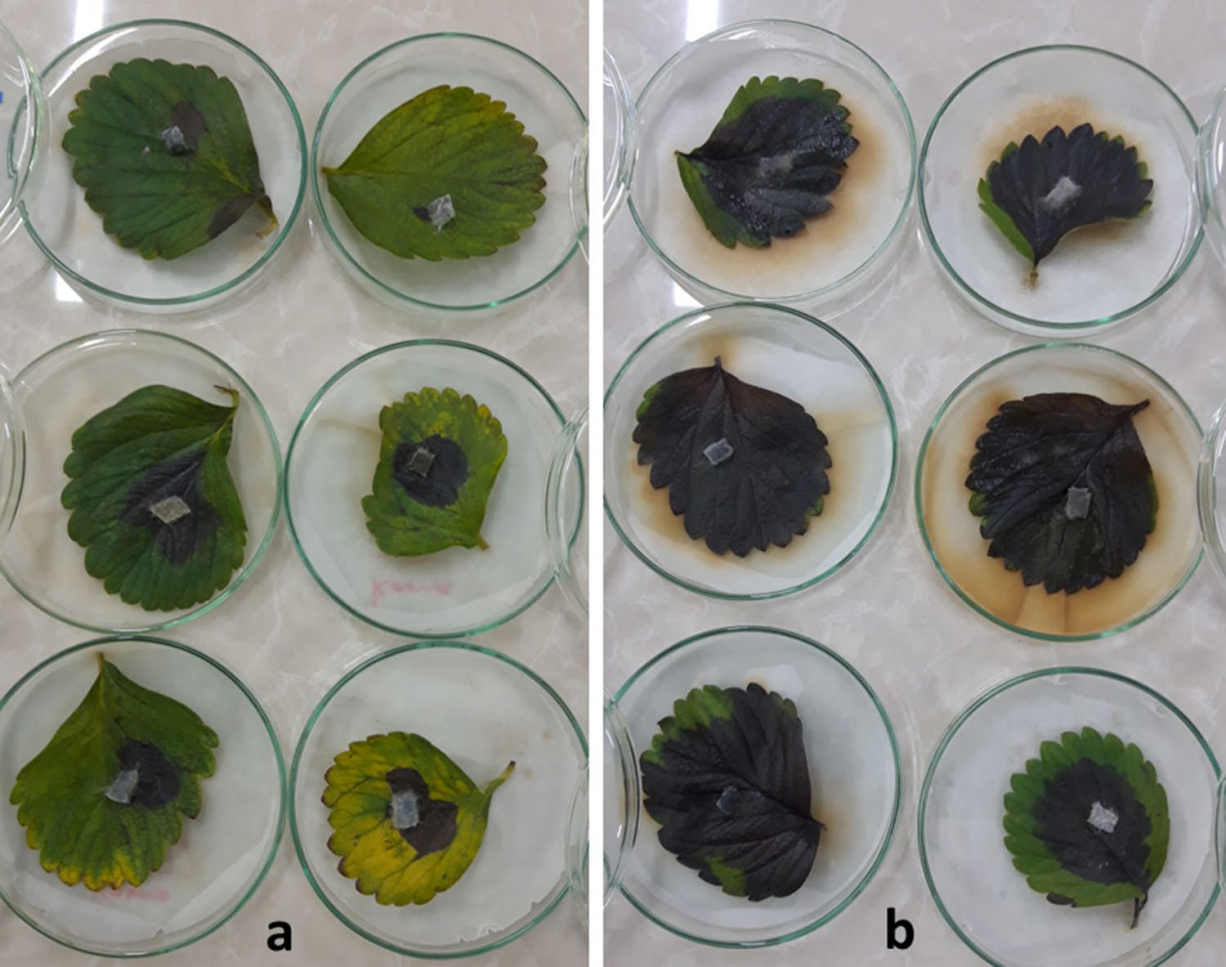
Bioassay of *pFGC-TEF-d* efficiency in the transient expression on *L. theobromae* in strawberry leaves (Camarosa) at 7 dpi. (**a**) Leaves transiently expressed *pFGC-TEF-d *as a treatment and (**b**) control leaves expressing empty plasmid (*pFGC*5941).

The necrotic spot appeared 3 dpi in both treatment and control of Camarosa strawberry cultivar transformed with recombinant (*pFGC-TEF-d*) and empty (*pFGC*5941) vectors, respectively. At 7 dpi, due to the spread of necrotic spots on the leaf surface, control leaves expressing *pFGC*5941 became completely necrotic. In treatment leaves expressing *pFGC-TEF-d* disease progress rate was significantly different from control leaves at *P* = 0.05 (Table 1, T-test score was 0.012). The statistical analysis using T-test showed the average size of the necrotic spots in treatment leaves (10 cm^2^) reduced by 58% compared to the control leaves (24 cm^2^) (Table 1 and Fig. 2).

**Table 1:** T- test analysis of transiently expressed *pFGC-TEF-d* on necrosis spot caused by *L. theobromae* using SPSS software in strawberry and grapevine.

	Repeat	Mean	Std. deviation	Std. error mean	T- test
**Strawberry Camarosa**					
c	6	24	3	1	0.012
t	6	10	9	3	
**Strawberry Ventana**					
c	6	14	0	0	0.00
t	6	1	1	0	
**Grapevine (Bidaneh Sefid)**					
c	10	27	9.06	2	0.00
t	10	12	4	1

**Figure 3:**
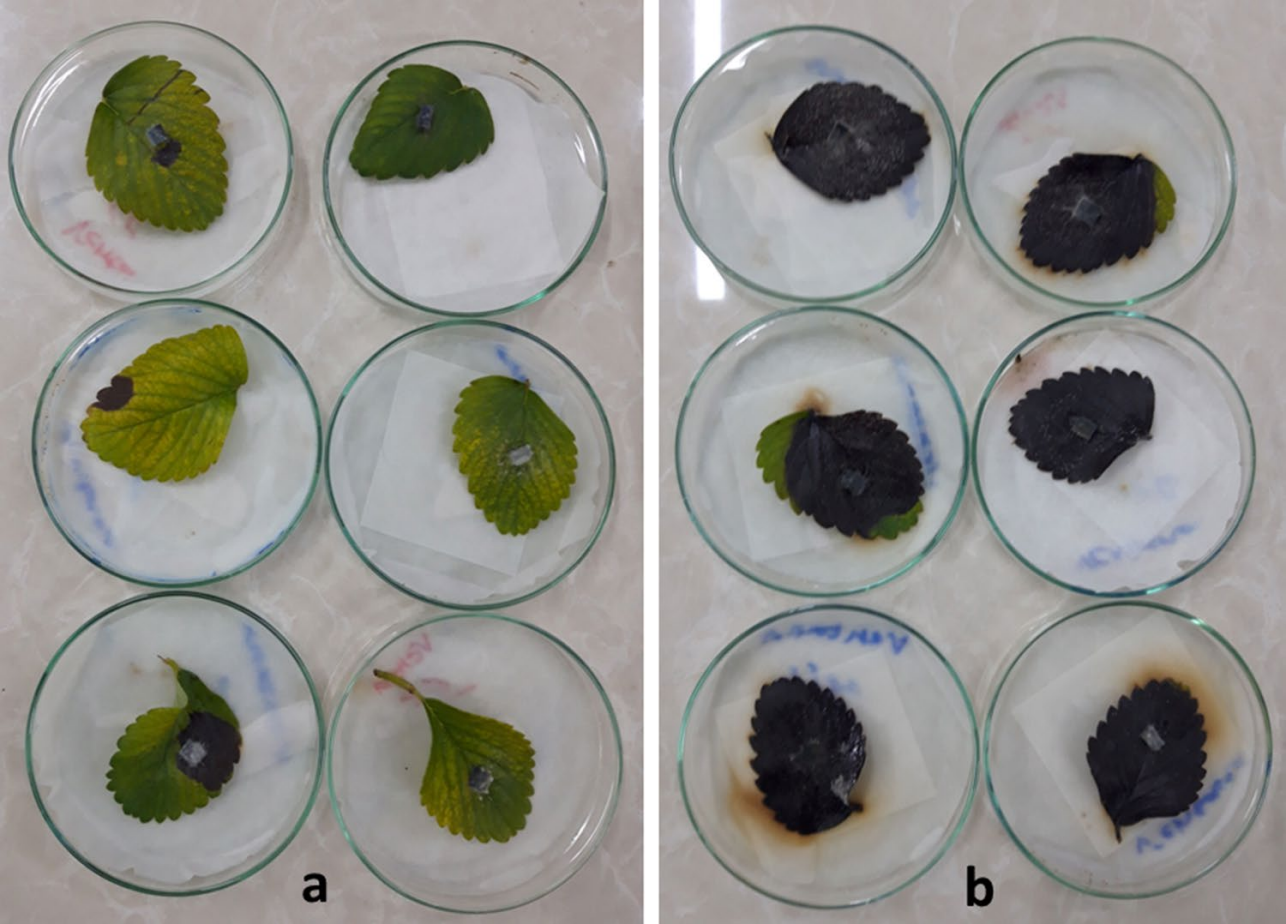
Bioassay of *pFGC-TEF-d* effects in control of *L. theobromae *in Ventana cultivar of strawberry leaves at 16 dpi. (**a**) Leaves transiently expressed *pFGC-TEF-d* as a treatment and (**b**) control leaves expressing empty plasmid (*pFGC*5941).

The analysis of construct efficiency on Ventana strawberry cultivar indicated a significant difference between treatment and control (*P* < 0.05). In Ventana cultivar, the necrotic spot appeared 8 and 5 dpi in treatment and control leaves, respectively. At 16 dpi, the whole surface of the control leaves covered by the necrotic spot, but not the treatment leaves and, a significant difference was observed between the mean size of necrotic spots in control (14 cm^2^) and treatment leaves (1 cm^2^) at this time point at *P* = 0.05 (Table 1, T-test score was 0.012) and necrotic spot diameter reduced by 93% in treatment leaves (Table 1 and Fig. 3).

**Figure 4:**
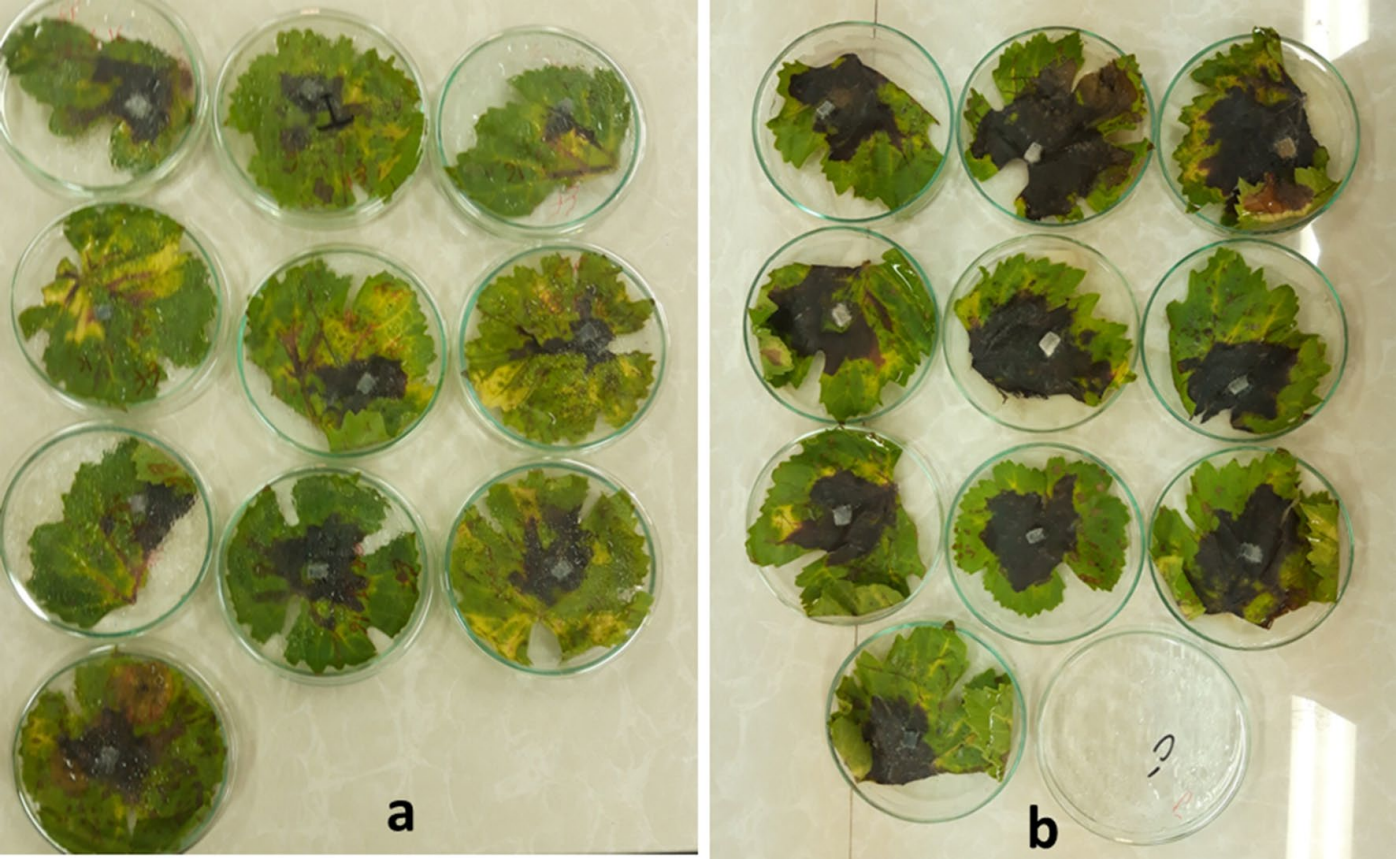
Bioassay of *pFGC-TEF-d* effects on necrosis 8 dpi by *L. theobromae* in grapevine (Bidaneh Sefid) leaves. (**a**) Leaves transiently expressed* pFGC-TEF-d *as a treatment and (**b**) control leaves expressing empty plasmid (*pFGC*5941).

Collected data from grapevine (Bidaneh Sefid cultivar) revealed the efficiency of *pFGC-TEF-d* construct in the reduction of necrotic spot diameter caused by *L. theobromae*. In the grapevine, the necrosis symptom appeared at 3 dpi. Although the necrosis spot appeared in all control and treatment leaves at 8 dpi, data analysis at this time showed a significant difference between control and treatment at *P* = 0.05 (Table 1). The mean size of necrotic spots in treatment leaves expressing *pFGC-TEF-d* construct (12 cm^2^) reduced by 55% compared to the control leaves (27 cm^2^, Fig. 4).

**Figure 5:**
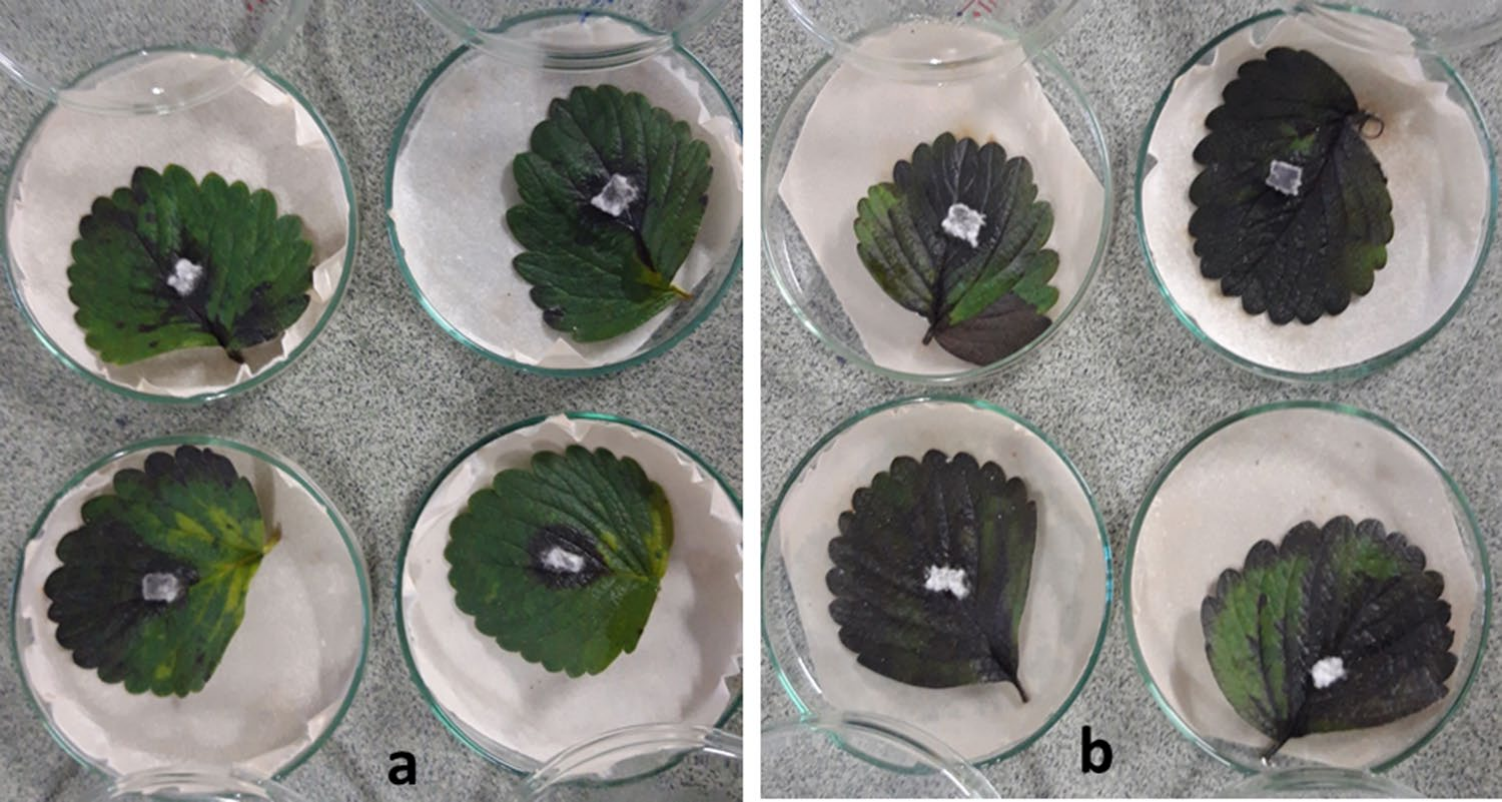
Necrosis symptoms on strawberry leave (Camarosa) 7 dpi by *N. parvum*. (**a**) Leaves transiently expressed *pFGC-TEF-d* as a treatment and (**b**) control leaves expressing empty plasmid (*pFGC*5941).

The efficiency of *pFGC-TEF-d* on *N. parvum* was evaluated on Camarosa strawberry cultivar. Disease symptom (necrotic spot) appeared at 4 dpi. At 7 dpi the control leaves were completely covered by necrotic spot (Fig. 5). Statistical analysis of the mean size of necrotic spots in control (23 cm^2^) and treatment leaves (7 cm^2^) showed a significant difference at *P* = 0.05. Data analysis at 7 dpi confirmed a reduction in disease progression in treatment leaves by 70% (Table 2 and Fig. 5). Despite low sequence similarity (72%) with *L. theobromae*, statistical analysis using T-test at 7 dpi indicated the efficiency of *pFGC-TEF-d* in control of *N. parvum*.

**Table 2:** Statistical analysis of transiently expressed *pFGC-TEF-d* on necrosis spot caused by *N. parvum *in strawberry (Camarosa cultivar) using T- test.

	Repeat	Mean	Std. deviation	Std. error mean	T- test
C	4	23	4	0	0.003
t	4	7	1	2	

### Detection of small RNAs in plants

**Figure 6:**
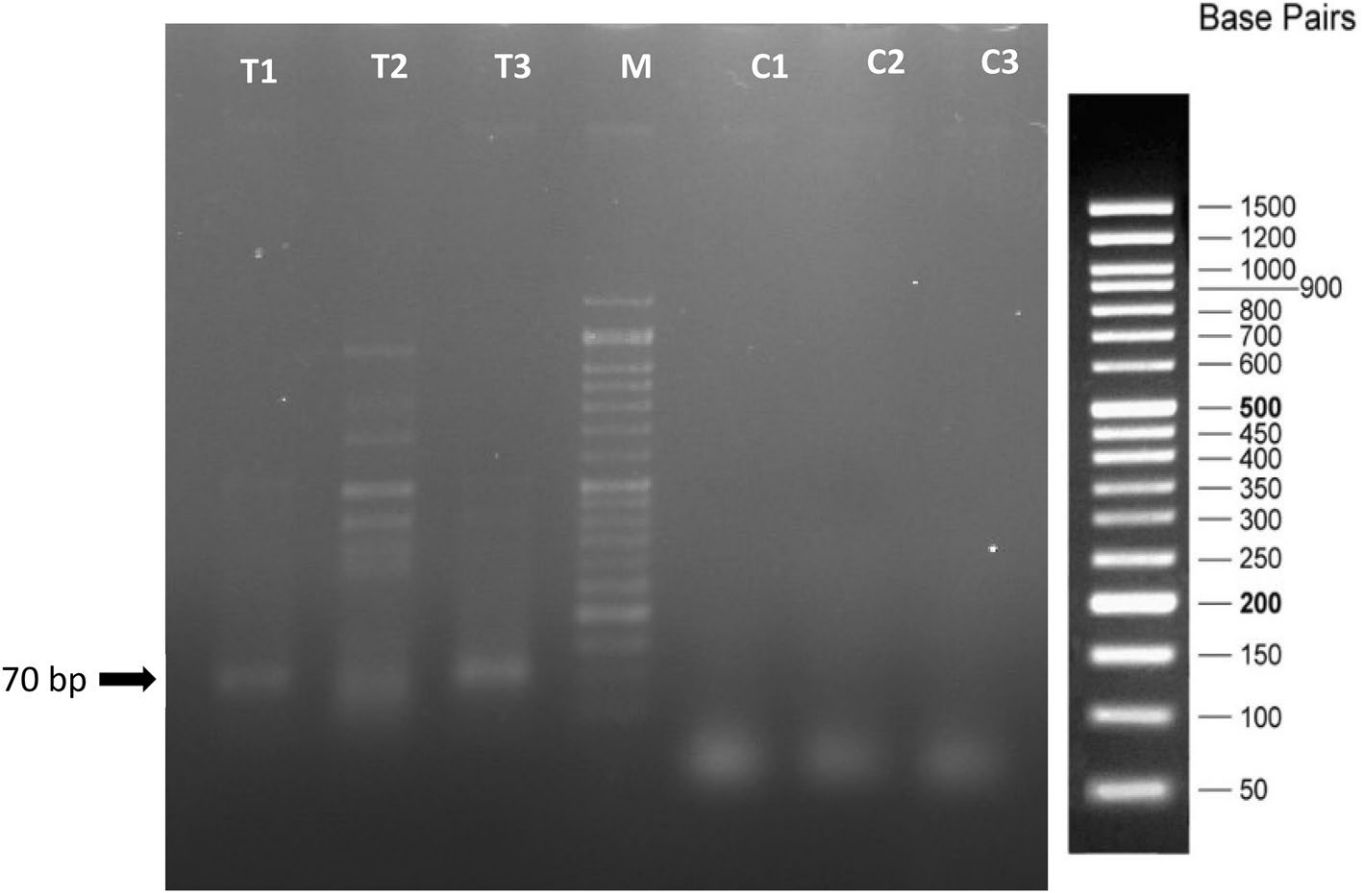
Detection of siRNA in strawberry plants expressing *pFGC-TEF-d* compare the control plants without *pFGC-TEF-d* expression. 70 bp shows siRNA detection. T1, T2, and T3 are replications of plants expressing pFGC-TEF-d. C1, C2, and C3 are control plants with no *pFGC-TEF-d* expression.

Detection of small RNAs performed using stem-loop PCR in strawberry plants transiently transformed using agrobacterium harboring *pFGC-TEF-d.* Stem-loop PCR results showed amplification of 90 bp band related to the small RNA and shown induction of small RNAs against *L. theobromae* and *N. parvum* in strawberry leaves. However, no bands were amplified in control plants transiently transformed using agrobacterium without *pFGC-TEF-d* (Fig. 6).

### qPCR of *Tef-1α*

**Figure 7:**
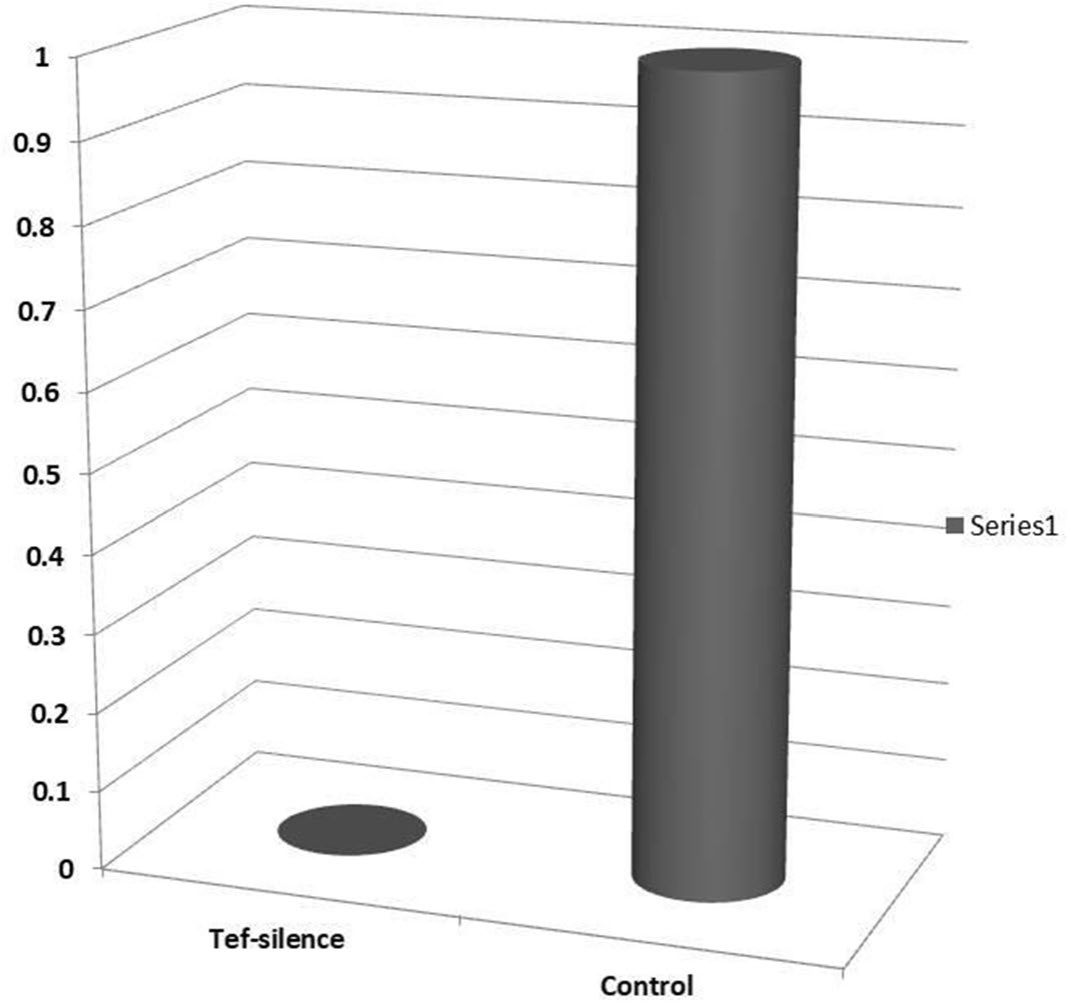
Comparative expression of *Tef-1α* in silenced and non-silenced *L. theobromae* using *pFGC-TEF-d*. Quantitative PCR showed a clear reduction of *Tef-1α *expression (518,134 fold) in silenced fungi using siRNA and hairpin structure of *Tef-1α* compare to the control samples.

Quantification of *Tef-1α* expression in treatment and control *L. theobromae* was performed using qRT-PCR. Analysis of CT of target and control genes in treatment and control *L. theobromae* indicated strong suppression of *Tef-1α* in treatment (518,134 Fold) in comparison to control samples (Fig. 7 and S5).

## Discussion

*L. theobromae* is an important worldwide necrotrophic phytopathogenic fungus reported from several plant species^1,3^ and *L. theobromae* is also known as a human opportunistic pathogen^7^. Over the years, management of the Botryosphaeriaceae members (e.g. *L. theobromae*) relied on chemical control, but today because of the concerns about the side effect of fungicides and chemical residues^14^, we need to pay significant attention to the green control of plant diseases. Antagonistic microorganisms (e.g. bacteria) can be used as an alternative and safe biological agents, but there are some drawbacks such as  dependency of these agents on ecological factors and though  application of some products of these agents improved biological control^27^, nowadays other safe and stable methods like genetically methods are desirable. Among several safe alternative methods recently considered, RNA silencing has received special attention^28,29^ and here we decided to examine the ability of this technology to the management of *L. theobromae* and *N. parvum.*

According to the previous studies, the expression of complementary hpRNA structures can efficiently repress the target sequences^30^. Thus, the aim of this study was to management of *L. theobromae* using a self-complementary hairpin structure. As a sequence region per se plays a role in the efficiency of gene construct^31^, we used the transient expression system using *Agrobacterium* to analyze the effectiveness of gene construct in the induction of resistance against *L. theobromae *and *N. parvum*. The *Agrobacterium*-mediated transient expression assay has developed as an easy and rapid method to analyze gene constructs in plants^32,33^. Increasing evidence about the role of *Agrobacterium* strain^34^, plasmid components such as promoter^31,34^, and host plants^34^, led us to transiently evaluate the efficiency of the designed construct before assay on transgenic plants. Given the fact that the selection of target sequences with the highly conserved regions is one of the preferable critical points, a conserved gene region of *L. theobromae* was selected to achieve a broad-spectrum resistance^35^. In recent decades, researchers have made efforts to improve the resistance efficiency of RNA silencing through choosing the best target sites^36^ and to achieve the best results, some protocols and critical points were published and emphasized^29,37^.

Therefore, we searched to find an efficient target site to control *L. theobromae*, a plant pathogen with a broad host range, and found *Tef-1α*, as a conserved gene in *L. theobromae* isolates with more than 96% similarity. Despite the frequent recommendations of RNAi technology for the management of plant diseases, one drawback associated with this technology is a generation of siRNAs that silence non-target genes^29^. However, no high similar (or close) target sites detected through in silico searching for off-targets in human and some important plant hosts (e.g. grapevine and strawberry). Furthermore, a significant role in fungal protein translation machine, two predicted microRNAs (Supplementary Fig S2 online) suggesting more putative biological role, and the existence of a single copy in fungal genome^38^, led to the selection of *Tef-1α* as a candidate gene.

Although, previous studies suggest that regions containing high secondary structure in RNA sequences are less accessible to siRNA and targeting sequences with the less structured area may show high resistance level^35^, in this research, we found that *Tef-1α* with high secondary structure can induce reasonable resistance against *L. theobromae* and *N. parvum*. In this survey, the candidate gene region was 316 bp in the range of recommended fragment sequences 200–400 bp^28^. It is important to note that shorter fragments are not efficient for induction of resistance and off-targets chance increases with longer sequences^28^. Since for each particular silencing construct experimental analysis requires^37^, the efficiency of the prepared construct was evaluated transiently in grapevine and strawberry as the important hosts, and also the down expression of *Tef-1α* in *L. theobromae* by inoculation with hairpin structure and small RNAs was evaluated.

As we can infer from the literature, resistance based on silencing can protect plants against related sequences in different pathogens^39^. Thus, we examined the induction of resistance against *Neofusicoccum* (*N. parvum*) as a closely related genus to the *Lasiodiplodia*. In spite of low sequence similarity (72%) with *L. theobromae* (Supplementary Fig S4 online), our results showed a reduction in disease progress caused by *N. parvum* (Fig. 5 and Table 2). Sequence alignment between the designed construct and *N. parvum Tef-1α* showed that *pFGC-TEF-d* can produce two predictable microRNAs against *N. parvum Tef-1α.* Therefore, the attachment of produced siRNA as microRNA to the *N. parvum Tef-1α* mRNA and also to the DNA sequence of *N. parvum Tef-1α* as a repressor of transcription may be the possible expectance for the induction of silencing against *N. parvum*.

Targeting multifunctional proteins (e.g. *Tef-1α*) as a candidate gene for silencing is an advantage^40^, because as previously indicated silencing of multifunctional genes can induce stable resistance against pathogens^39^.

Considering the successful performance of hairpin RNA in the previous studies, comparing the sense and antisense RNAs^41,42^ in this research hairpin structure was developed for control of *L. theobromae*. Our findings on the necrotrophic fungus *L. theobromae* showed RNA silencing can be a new prospect technology for control of necrotrophic pathogens as biotrophs and this finding are in line with Andrade et al.^32^, who showed effective control of *Sclerotinia sclerotiorum* using gene silencing. Using hairpin structure for silencing of *chitin synthase* (*chs*) in necrotrophic fungus, *Sclerotinia sclerotiorum,* showed 55.5–86.7% reduction in disease severity in transgenic tobacco plants 72 h post-inoculation^32^.

According to Tan et al.^34^, who mentioned that the efficiency of gene constructs may depend on the host genetic background, our results revealed that the occurrence of disease symptoms depends on the cultivar’s genetic background as disease symptoms observed 3 dpi in strawberry Camarosa cultivar compare to 8 dpi in Ventana cultivar.

Induction of small RNAs in strawberry plants expressing *pFGC-TEF-d* improved 3 days after syringe infiltration using specific stem-loop PCR (Fig. 6), thus we confirmed the expression and production of hairpin structure and small RNAs.

The time interval between agro-infiltration and pathogen inoculation showed as an important parameter for the efficiency of silencing construct^41,43^, and as in this study, we detected induction of siRNAs in plants 3 days after transient expression, therefore, in this study we inoculated leaves by fungal pathogen 3 days after agro-infiltration to allow the accumulation of siRNAs before the fungal inoculation. As the results indicated, the *pFGC-TEF-d* cannot prevent the disease initiation, but decreases the disease progress as expected based on the function of the targeted gene *Tef-1α*. Quantitative real-time PCR was conducted for evaluation of downregulation of *Tef-1α* in *L. theobromae* using specific primers and results showed strong suppression of *Tef-1α* in *L. theobromae* treated with total RNA extracted from *N. benthamiana* transiently expressing *pFGC-TEF-d* (Fig. 7)*.* Based on our knowledge, this is the first study using *Tef-1α* for control of plant pathogenic fungi using RNA silencing technology. This study showed that the gene construct designed based on *L. theobromae Tef-1α* (*pFGC-TEF-d*) is able to control not only *L. theobromae*, but is also able to control of *N. parvum* another species belong to the same family Botryosphaeriaceae.

Although the mechanisms of small RNAs transport from plants to fungal pathogens its not completely, understood some studies have documented^44^. small RNAs trafficking from Arabidopsis to *Botrytis cinerea* using plant extracellular vesicles ^44^. Thus, trafficking of siRNAs from host plants to the fungal pathogens plays a vital role in the induction of resistance to pathogens^45^. Though there is contradictory results in *Colletotrichum gloeosporioides* regarding the ability of fungal pathogen to uptake RNA molecules from host plants ^45,46^, and there is no evidence for small RNAs transport in Zymoseptoria tritici–wheat pathosystem^46,47^. Topical application of dsRNAs targeting plant pathogenic fungi showed most fungi can uptake stable dsRNAs efficiently^48^. However, in our study in vitro analysis of targeted gene using RT- qPCR in *L. teobromae* showed a significant decrease in *Tef-1α* transcript (tends to zero) (Fig. 7*),* indicating the absorption of small RNAs or long hairpin RNAs by *L. theobromae*, but it is not clear which form of RNA molecule can uptake by examined fungi.

Finally in this study, we introduce an efficient silencing construct against *L. theomobrae* and* N. parvum* to develop transgenic plants or for exogenous application as it is used for some other pathogens^35^, especially for countries that GMO is not allowed. To determine the performance rate of the siRNA technology to management of botryosphaeriaceous fungi we recommend to investigate the effect of *pFGC-TEF-d* construct on closely related pathogens from Botryospaheriaceae on different hosts in future studies.

## Material and methods

### Fungal strains and experimental plants

*L. theobromae* (IRAN 1499C; GU973861) isolated from mango (*Mangifera indica*) and *Neofusicoccum parvum* (IRAN 1535C; JQ772082) isolated from white willow (*Salix *sp.) were obtained from the Mycology lab, Department of Plant Protection, University of Kurdistan^3,48^. The experiment was conducted in 2019 and 2020. The efficiency of the construct was examined on the grapevine (Bidaneh Sefid cultivar) and strawberry (Camarosa and Ventana cultivars) from the Strawberry Research Center, University of Kurdistan.

### DNA extraction

DNA extraction was carried out according to the modified method of Raeder and Broda (1985) as described by Abdollahzadeh et al.^49^.

### A selected gene of interest

The *Tef-1α* gene contains a conserved region in *L. theobromae* population was selected as a candidate gene (MG192354.1). The conservation of gene sequence was checked in GenBank, NCBI. In silico analysis revealed that the selected gene shows a high intra-species but low inter-species similarity. Analysis of off-target in plants and human sequences was investigated using RNAi scan (http://bioinfo2.noble.org/cgi-bin/RNAiScan/RNAiScan.pl) and [plantgrn.noble.org/pssRNAit/]. Secondary structure of RNA predicted using RNA structure software and predicted miRNA from this gene revealed using Evry RNA-miRNAFold online software.

### Cloning

When the target sequence is selected, specific primers (Supplementary Table S1 online) are designed for amplification of the partial *Tef-1α* gene. In a primer pair, the restriction enzyme site sequences and three nucleotides as an anchor were considered. PCR amplification was carried out using La.TEF1-α-F and La.TEF1-α-R primers in Biorad (T100TM) thermal cycler. PCR condition was as follows: 94 °C for 5 min; 30 cycles of 94 °C for 30 S, 54 °C for 1 min, 72 °C for 1 min; and a final extension of 72 °C for 10 min. PCR product was loaded in 1.2% agarose gel with 0.5X TBE buffer. PCR product (3 µl, 316 bp) (Supplementary Fig S1a online) was digested using *Nco*I restriction enzyme and after digestion, the product was purified using Favoregen (Taiwan) kit. The digested product was self-ligated in 10 µl reaction by T4 ligase (100 ng of DNA, 1 µl of 10X ligase buffer, and 100 U of T4 ligase) for 1 h at 22 °C and overnight at 4 °C.

The ligation reaction was performed as a template for amplification of *Tef-1α* dimer using TEF1-α primer with the same PCR amplification condition. The amplified dimer was cloned in pTG19-T vector (Vivantis, Malaysia) using T4 ligase (100 U T4 ligase, 25 ng pTG19-T, 100 ng PCR products, and 1 µl of 10X ligase buffer) for 1 h in 22 °C and overnight in 4 °C. The ligation reaction product transformed to *E. coli* DH5α using heat shock method^50^. The plasmid was extracted from the white clone and then the recombinant plasmid was confirmed by digestion. Thereupon, the recombinant pTG19 was digested for separation of *tef1-α* dimer using *Xba*I and *Xho*I enzymes. The digestion product loaded on 1% agarose gel and the dimer band was purified using FavorPrep™ GEL/ PCR Purification Kit (Favoregene, UK). Moreover, the digestion of *pFGC*5941 binary vector done using the same enzymes, and the plasmid backbone was purified on the agarose gel. Insertion of *tef1-α* dimer into *pFGC*5941 was done using ligation reaction containing 1 µl 10X ligation buffer, 100 ng *tef1-α* dimer, 50 ng digested *pFGC*5941, and T4 ligase (100 U) in a 10 µl reaction volume. Again, the ligation product was transformed to *E. coli* DH5α using the heat shock method and plasmid extraction was carried out using alkaline lysis protocol^51^. Moreover, recombination of *pFGC*5941 was performed using clone PCR, endonuclease restriction digestion, and sequencing. The cloning steps are shown in Fig. 1.

### Agrobacterium transformation

*Agrobacterium* LBA 4404 strain was cultured in LB medium containing rifampicin for 48 h, at 28 °C /150 rpm. Then, the bacterial suspension precipitated in a 2 ml tube using a centrifuge (5000 rpm, 3 min). The precipitated bacterial cell was re-suspended in 250 µl TE buffer, washed, and then centrifuged. The bacterial cell pellet was re-suspended in 250 µl of LB with 0.1 concentrations, and then the tube was put in liquid nitrogen for seconds and kept on ice for 30 min. Then, 2 µl of the binary vector (100 ng/µl) was added and the tube was put in liquid nitrogen for seconds and after that, the sample was kept at 37 °C for five minutes. Finally, for recovery, sample tubes were kept on a shaker incubator at room temperature (25–28 °C, 180 rpm) for 4 h. Finally, 200 µl of the cell suspension was spread on selective LB media containing rifampicin and kanamycin.

### Transient expression

Grape (Bidaneh Sefid cultivar) and strawberry leaves (Camarosa and Ventana cultivars) were infiltrated with *Agrobacterium tumefaciens* LBA4404 isolate using vacuum-based infiltration as described by Kapila et al.^52^. For infiltration, the 2-day-old Agrobacterium cell suspension (OD600 = 0.8) was centrifuged (5000 rpm, 3 min, 4 °C) and the harvested cells were re-suspended in sterile water and kept on ice for use. Plant leaves were plunged in cell suspension under vacuum pressure to penetration of cell suspension into the mesophyll cells. When the majority part of the leaves was soaked, the leaves were transferred to the petri dishes containing soaked sterile paper. The petri dishes were incubated in a growth chamber (16/8 h light/night regime, 25 °C). Each experiment repeated two times and in each time 6–10 leaves were used as a biological replication and. *A. tumefaciens* LBA4404 containing empty p*FGC*5941 and *pFGC-TEF-d* used as control and treatment, respectively.

### Fungal inoculation

Three days after vacuum infiltration, a small disc (0.5 × 0.5 cm) of 4 day-old fungal colony on potato dextrose agar (PDA) was placed in the middle of each leaves incubated in the growth chamber (16/8 h light/dark regime, 25 °C). Data were recorded until the end of the experiments for three weeks.

### Evaluation of resistance

Resistance evaluation of vacuum infiltrated leaves with Agrobacterium suspension (OD_600_ = 0.3) was begun three days after inoculation with a fungal disc, until the necrotic spot covered whole leaves in control plants (7–20 days after inoculation, depends on the host responses). Resistance evaluation analysis was performed based on the necrotic spot diameter. The experiment was replicated 2 times.

### Induction of small RNAs and RNA extraction from Strawberry plants

For detection of small RNAs in strawberry plants, agrobacterium suspension (OD_600_ = 0.3) harboring *pFGC-TEF-d* vector was injected under the epidermis of three strawberry leaves as a treatment and agrobacterium suspension without the vector was injected into the leaves of 3 other strawberry plant as a control. Three days after expression, total RNA was extracted from each sample and used for stem-loop PCR using specific primers. Total RNA extraction from strawberry leaves was extracted according to the protocol developed by Fusaro et al.^23^.

## Detection of small RNA

Small RNA detection was done using stem-loop PCR. For detection of siRNA, cDNA was synthesized using the stem-loop primer (Table S1) and easy cDNA synthesized kit (Pars tous, Iran). Reverse transcription reactions were done in 10 µl containing 3 µl of RNA (50 µg/ml) treated with DNase, 1 µl of Enzyme mix, 5 µl of buffer mix, and 1 µl of DEPC water. Microtubes were kept at 16 °C for 15 min,15 min at 25 °C, 30 min at 47 °C, and then the reactions were stopped at 70 °C for 10 min. PCR amplification was done in 12 µl reaction containing 6 µl of 2X Master mix PCR buffer, 1 µl of cDNA, and 0.5 µl (10 pmol/µl) of Tef-micro-F and Tef-micro-R primers (Table S1).

### Generation of hpRNA and siRNA

The agrobacterium containing *pFGC-TEF-d* (OD_600_ = 0.3) was infiltrated under the epidermis of *Nicotiana benthamiana* leaves using a syringe and after 3 days the total RNA was extracted from inoculated leaves using the protocol developed by^53^, and used as a source of hpRNA and siRNA for induction of silencing in *L. theobromae*.

### Analysis of *Tef-1α* downregulation and Fungi RNA extraction

Analysis of down-regulation of the target gene was performed in liquid cultured (PD media) *L. theobromae*. The *L. theobromae* fungus was grown in 20 ml potato dextrose media in a shaker incubator with 200 rpm and 25 °C for 3 days. After 3 days 350 µl of 2 µg/µl total RNA extracted from *N. benthamiana* was added to the medium and allowed to grow under the same condition for two more days. Two days post-induction of silencing, total RNA was extracted from fungal mycelia using the protocol developed by Sánchez-Rodríguez et al.^54^. RNA was extracted from *L. theobromae* cultured in PD media without adding RNA as a control.

### Quantitative real-time PCR

Comparative expression of *Tef-1α* was carried out by real-time PCR using Tef-real time-F and Tef-real time-R as a target and IRCD-F and IRCD-R as an internal control primers (Table S1). Complementary cDNA was synthesized using RNAs extracted from *L. theobromae* using random hexamer in reactions as mentioned above and cDNA synthesis checked using PCR (Fig S5). Real-time PCR was carried out in 10 µl reaction containing 5 µl qPCR master mix (RealQ Plus Master Mix Green, Ampliqon, Denmark), 3 µl of RNA (16 ng/µl), 0.5 µl of forward and reverse primers (10 ng/µl) and 1 µl of DEPC water. The number of biological replication was 3 and technical replication was 2.

### Statistical analysis

Collected data subjected to T-test statistical comparison analysis using SPSS (SPSS Statistics Version 22) software.

## Supplementary Information

Supplementary Information.
