# Supplementary material for: Development of an efficient Tef-1α RNA hairpin structure to efficient management of Lasiodiplodia theobromae and Neofusicoccum parvum
Source: Sci Rep. 2021 May 5;11:9612. doi: 10.1038/s41598-021-88422-1 (PMC8099910; doi:10.1038/s41598-021-88422-1)
Supplement: Supplementary file 1 — Supplementary Information. [file 41598_2021_88422_MOESM1_ESM.docx]

**Development of an efficient tef-1α RNA hairpin structure to efficient management of *Lasiodiplodia theobromae* and *Neofusicoccum parvum***

**Omid Nili^1^, Abdolbaset Azizi^1^*, Jafar Abdollahzadeh^1^***

1- Department of Plant Protection, University of Kurdistan, Sanandaj, Iran

*Corresponding authors: A.azizi@uok.ac.ir

J.abdollahzadeh@uok.ac.ir

Postal Code: 66177-15175

**Supplementary Information**

**Table S1: Primers were used in this research. Underline sequences represent the restriction enzyme sites.**

| Name | Sequences (5՜- 3՜) | Ann. Tm (C˚) | | Size (bp) |
| --- | --- | --- | --- | --- |
| La.TEF1-α-F | cgctctaga*ctcgag* catcgagaagttcgagaagg | 54 | | 316 |
| La.TEF1-α-R | ggcggatcc ccatggtacttgaaggaacccttacc |  |  |  |
| TEF1-α | catcgagaagttcgagaagg | 54 | | 610 |
| TA-f | acgctcgag gagctcggtacccgggga | 54 | | 650 |
| TA-r | ggtcgactctagaggcta |  |  |  |
| Tef-micro-F | gggcgcgatcggagcaga | 55 | 70 | |
| Tef-micro-R | ctggtgcagggtccgagg |  |  |  |
| Tef-Stem-loop | gttggctctggtgcagggtccgaggtattcgcaccagagccaaccggctt | | |  |
| Tef-real time-F | agtccgccttatcgctttc | 53 | 92 | |
| Tef-real time-R | caagtgcggtcattttgcc |  |  |  |
| IRCD-F | cctacagcaccaacactcag | 53 | 128 | |
| IRCD-R | agaagatgccgtcagaaacc |  |  |  |

ccatgg: *Nco*I ggatcc: *BamH*I tctaga: *Xba*I ctcgag: *Xho*I

**Table S2) Predicted *L. theobromae* *Tef-1α* efficient siRNAs using RNAi Scan**

| Number | Fragment | siRNA AS (3' - 5') |
| --- | --- | --- |
| 1 | query_seq (22 - 42) | GGCACGUGCGUACAGCAAAAA |
| 2 | query_seq (23 - 43) | GCACGUGCGUACAGCAAAAAA |
| 3 | query_seq (24 - 44) | CACGUGCGUACAGCAAAAAAU |
| 4 | query_seq (95 - 115) | GCGAAACCACUCCCCGUAAAA |
| 5 | query_seq (96 - 116) | CGAAACCACUCCCCGUAAAAA |
| 6 | query_seq (143 - 163) | GAGCAGACCCAAGCCGUUUUA |
| 7 | query_seq (145 - 165) | GCAGACCCAAGCCGUUUUACU |
| 8 | query_seq (158 - 178) | GUUUUACUGGCGUGAACCAAA |
| 9 | query_seq (204 - 224) | GCGGAGGGGUGAUCGCUUUUA |
| 10 | query_seq (207 - 227) | GAGGGGUGAUCGCUUUUACGA |


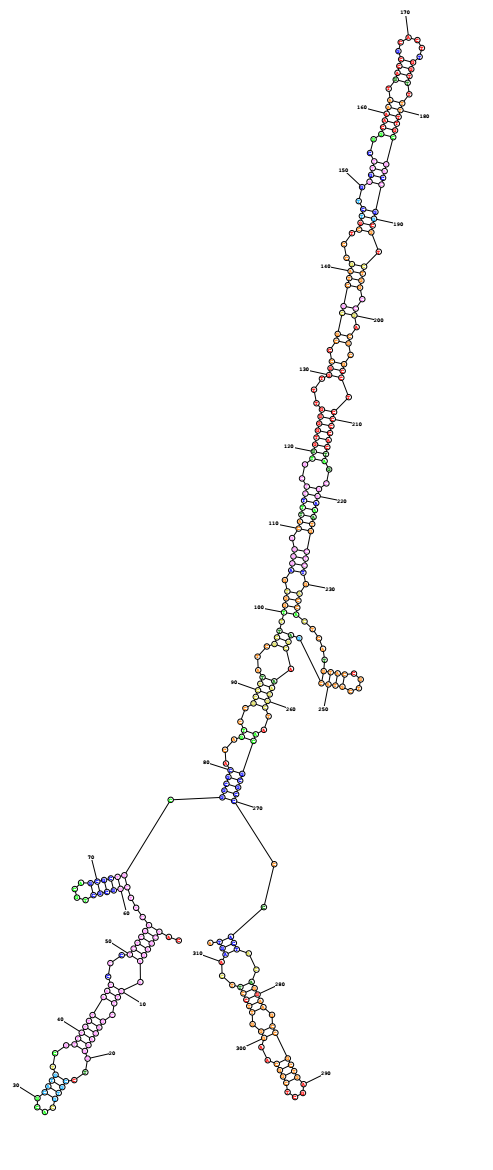


Fig S1): Prediction of the *L. theobromae* *Tef-1α* RNA secondary structure. The secondary structure was predicted using the Evry RNA-miRNAFold online software.


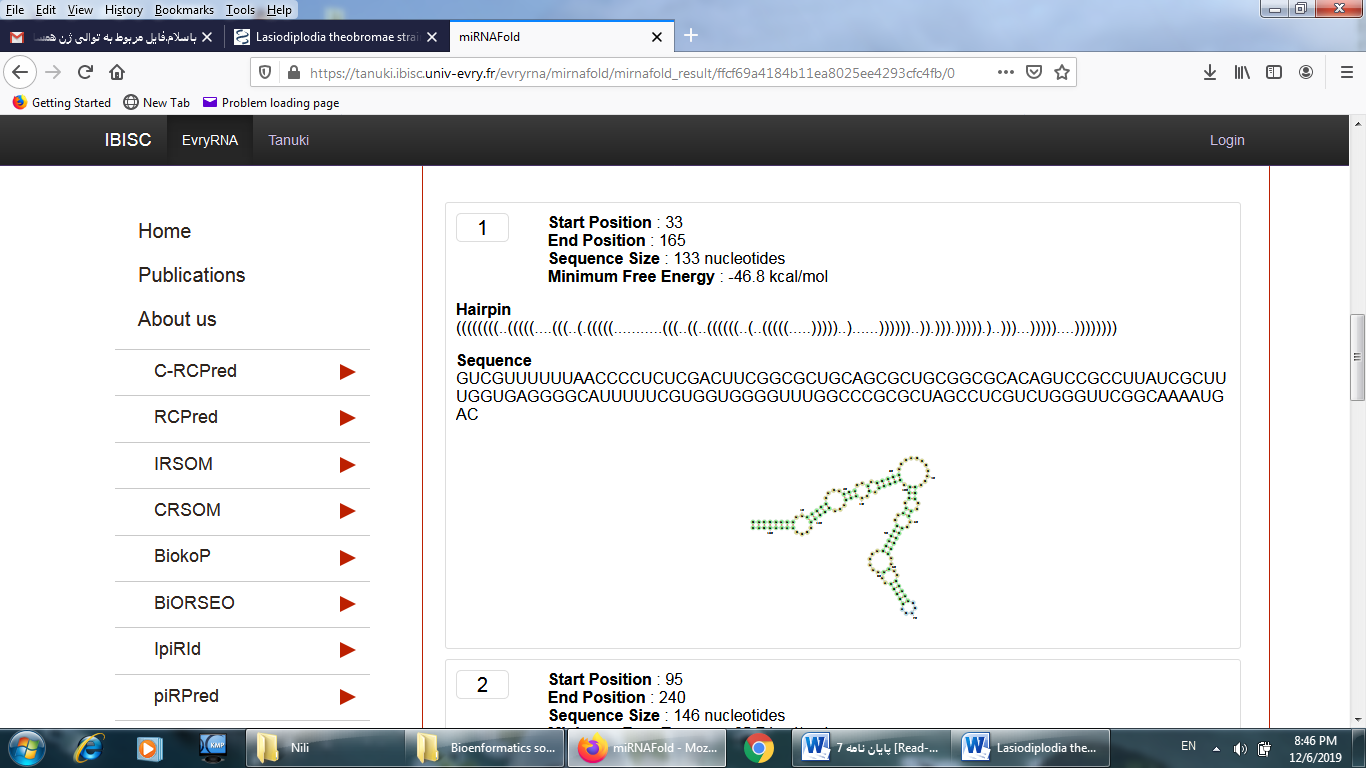


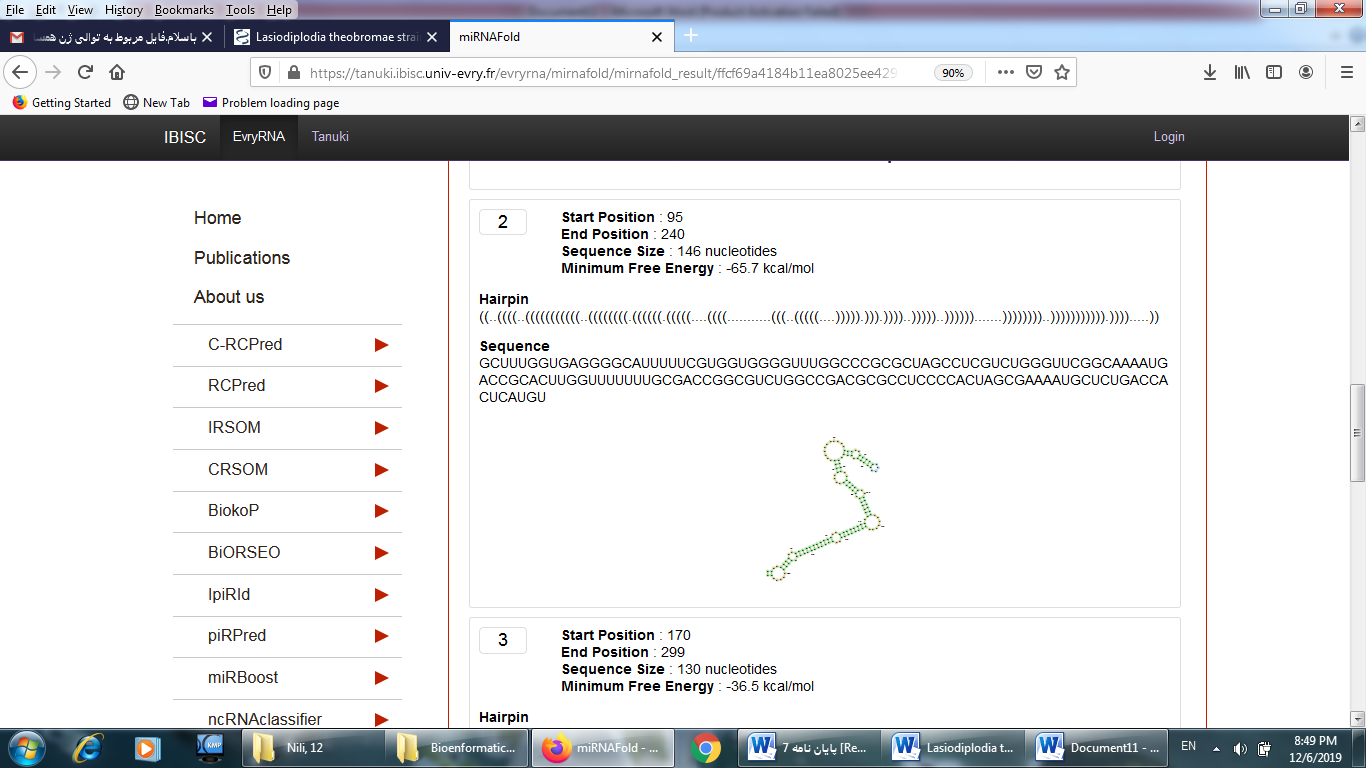


Fig S2) Predicted biogenesis of two microRNAs from *L. theobromae* *Tef-1α* using the Evry RNA-miRNAFold online software.


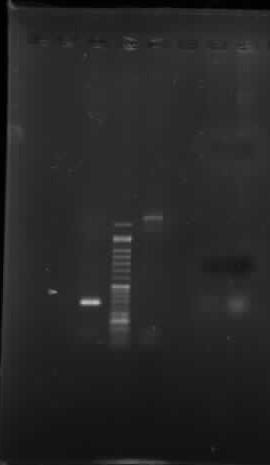

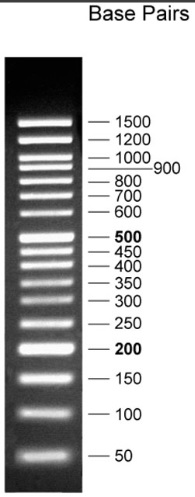


**1**

**2**

S3 a) PCR amplification *L. theobromae* *Tef-1α*. 316 bp *Tef-1α* fragment amplified using La.TEF1-α-R and TEF1-α-F primers from *L. theobromae* DNA. 1) *Tef-1α* amplified from *L. theobromae* 2) 100 bp marker


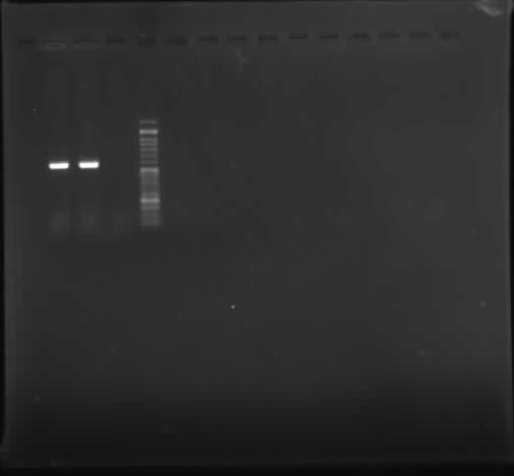

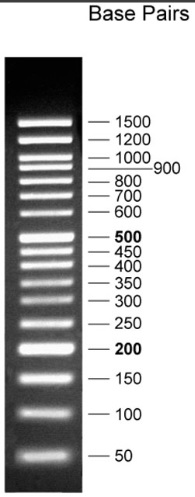


**1**

**2**

**3**

**4**

S3b) A 600 bp *Tef-1α* dimer fragment amplified from ligation products using TEF1-α-F primer. 1 and 2) Amplified band from ligation product 3) Negative control 4) 100 bp marker


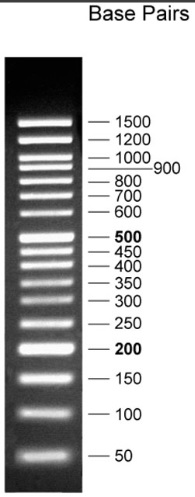

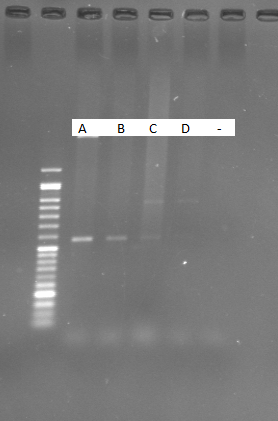


M

S3c) A dimer fragments amplified from clone PCR of *pFGC-TEF-d*. M) 100 bp marker a, b, c and d) clone PCR from some colony -) Negative PCR


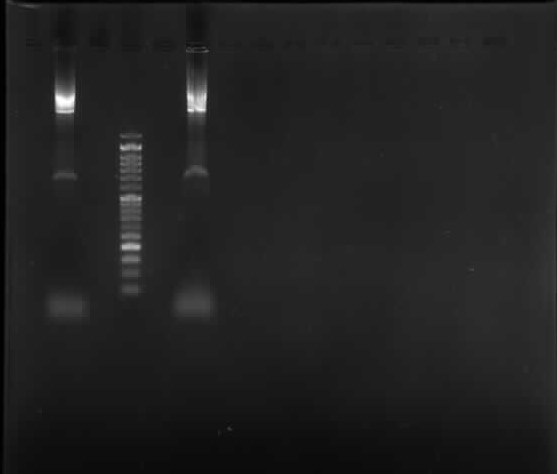

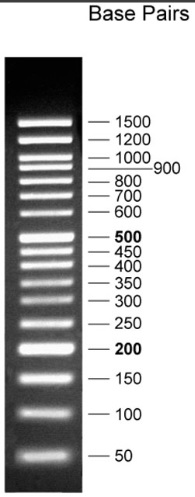


**4**

**1**

**2**

**3**

**5**

S3d) Digestion result using *BamH*I showed cloning of *Tef-1α* dimer into *pFGC5941* binary vector and development of *pFGC-TEF-d*. 1 and 5) recombinant plasmid digested by *BamH*I 3) 100 bp marker.


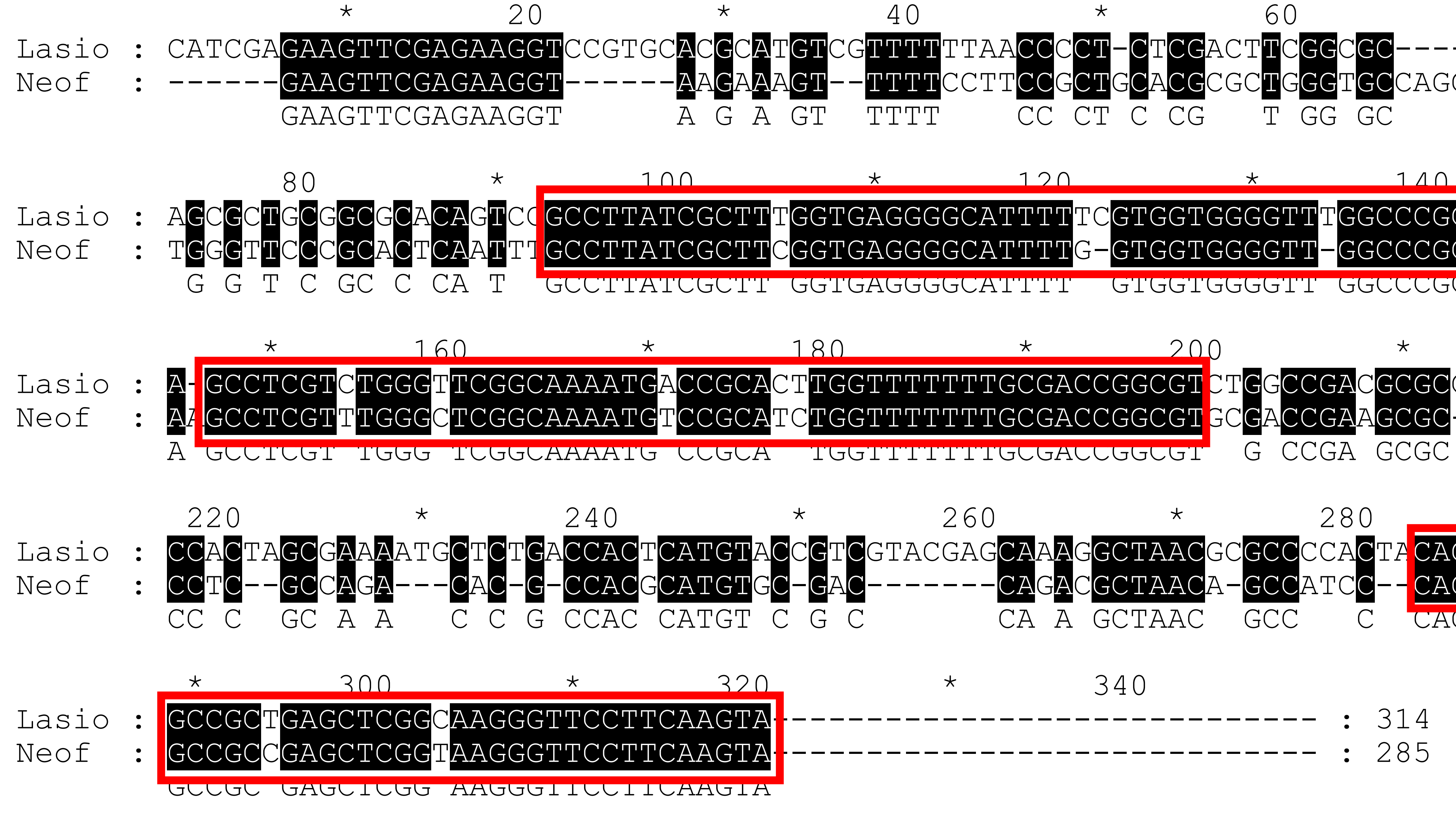


Fig S4) Sequence alignment of *L. theobromae* (MG192354.1) and *N. parvum* (JQ772082.1) *Tef-1α*. Alignment shows low sequence similarity (72%). Red boxes show sequences for siRNAs that may silence *N. parvum* *Tef-1α*.

**1**

**2**

**3**

**M**


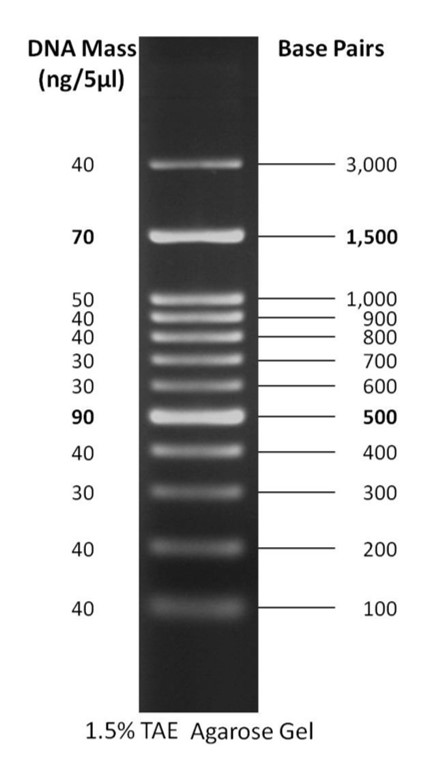


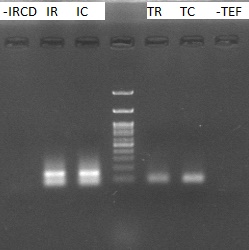


**T1**

**T2**

**T1**

**C1**

**C2**

**C3**

**M**

**IRCD**

**-IRCD**

Fig S5) Detection and optimize primer annealing temperature for real-time PCR. M) 100 bp marler, IRCD) Internal control gene TEF) *Tef-1α* NC) Negative control
